# Small extracellular vesicles‐based cell‐free strategies for therapy

**DOI:** 10.1002/mco2.57

**Published:** 2021-02-18

**Authors:** Yeye Guo, Huaishan Wang, Lili Huang, Lingling Ou, Jinjin Zhu, Shujing Liu, Xiaowei Xu

**Affiliations:** ^1^ Department of Pathology and Laboratory Medicine, Perelman School of Medicine University of Pennsylvania Philadelphia Pennsylvania USA

**Keywords:** cancer, exosome, extracellular vesicle, immunotherapy

## Abstract

Small extracellular vesicles (sEVs) are extracellular nanovesicles that contain bioactive proteins, lipids, RNA, and DNA. A variety of biological process is regulated with sEVs. sEVs are an intercellular messenger regulating recipient cell function and play a role in disease initiation and progression. sEVs derived from certain cells, such as mesenchymal stem cells and immune cells, have the potential for clinical therapy as they possess the characteristics of their parental cells. With better understanding of sEVs biogenesis, their transportation properties, extended circulatory capability, and exceptional biocompatibility, sEVs emerge as a potential therapeutic tool in the clinic. Here, we summarize applications of sEVs‐based therapies in different diseases and current knowledge about the strategies in bioengineered sEVs, as well as the challenges for their use in clinical settings.

## Graphical abstract

Small extracellular vesicles (sEVs) are extracellular nanovesicles that contain bioactive proteins, lipids, RNA, and DNA. A variety of biological process is regulated with sEVs. sEVs are an intercellular messenger regulating recipient cell function and play a role in disease initiation and progression. sEVs derived from certain cells, such as mesenchymal stem cells and immune cells, have the potential for clinical therapy as they possess the characteristics of their parental cells. With better understanding of sEVs biogenesis, their transportation properties, extended circulatory capability, and exceptional biocompatibility, sEVs emerge as a potential therapeutic tool in the clinic. Here, we summarize applications of sEVs‐based therapy in different diseases and current knowledge about the strategies in bioengineered sEVs, as well as the challenges for their use in clinical settings.

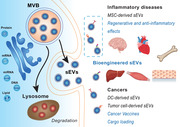

## 1 INTRODUCTION

Small extracellular vesicles (sEVs) are 30–200 nm lipid bilayer‐enclosed extracellular vesicles. A heterogenous mixture of sEV subsets, including exosomes, is derived when commonly used “exosome” isolation techniques are employed. Almost all cells generate sEVs and deliver to the surrounding biological fluids.^1^, ^2^ Briefly, the formation of sEVs is initiated from endosomal system. Early endosomes mature into late endosomes or multivesicular bodies (MVB) by inward budding of the endosomal membrane. Constitutively, the intraluminal vesicles (ILVs) within large MVBs are formed during this process that the endosomal membrane invaginates. When fusion with the plasma membrane, most ILVs are released into the extracellular space, and turn to sEVs.^3^, ^4^, ^5^

Small EVs were initially regarded as cellular garbage disposals as a result of cell damage, or by‐products of cell imbalance. Further studies found that those sEVs exhibited biological function. The bioactive cargo contains lots of information from their parental cell, including noncoding RNAs and mRNA,^6^ free fatty acids,^7^ surface receptors, and proteins^8^ inside and on the surface. Therefore, sEVs mediate the short‐range and distant communications between cells. Various target cells can be stimulated by the membrane molecules on sEVs or the contents inside sEVs.^9^ During tumor progression, sEVs from tumor cells may help to form a premetastatic niche for tumor metastasis.^10^

Over the past decades, many studies demonstrated that sEVs were associated with various diseases, such as inflammatory diseases,^11^ diabetes,^12^ cardiovascular diseases,^13^ central nervous system diseases,^14^ tumors,^15^ and so on. We recently showed that PD‐L1 on sEVs may be used to predict melanoma patient response to anti‐PD1 therapy.^16^

## 2 ADVANTAGES OF sEVs FOR THERAPY

With better understanding of sEVs, its application in clinical treatment attracts researchers’ attention. Compared to traditional drug delivery system or cellular therapy, sEVs possess some natural advantages such as they are biocompatible and biodegradable, therefore are low cytotoxic and immunogenic.^17^ Because of their tiny size, sEVs can be delivered to long‐distance sites and are able to escape from lung clearance and cross many biological barriers such as pass through the blood–brain barrier.^18^ Considering the natural transportation feature and long‐term circulatory capability, sEVs are ideal for carrying drugs, proteins, nucleic acids, and other objects for therapy.^19^, ^20^, ^21^ Another important characteristic of sEVs is the high specificity for target cells. It has been identified that sEVs origin could determine cell targeting and the transfer of chemicals toward target cells.^22^ In addition, sEVs are simple to produce as most cell types can produce sEVs and they can be stored easily.^23^ Taken together, these characteristics enable sEVs to be a promising candidate for therapy.

## 3 THE CURRENT STATE‐OF‐THE‐ART METHODS IN ISOLATION OF sEVs

Isolation of sEVs is labor intensive and is the bottleneck for using sEVs‐based diagnosis or treatment clinically. Ultracentrifugation is still the most common approach, which sometimes is combined with density gradient centrifugation. This is ideal for isolation of exosomes from large volume media. However, the process takes a long time and requires expensive instruments and good technical skill of researchers. The combination of ultracentrifugation with density gradient centrifugation helps to achieve a higher purity of sEVs.^24^ Isolation kits based on size and membrane‐based affinity binding have been launched by some companies.^25^ Precipitation of sEVs is another popular way for isolating sEVs especially from body fluids.^26^ These approaches provide a choice to isolate sEVs easily in lab. For research requiring downstream analysis, magnetic bead‐based isolation kit is a favorable manner.^27^ With these options, investigators can choose one of the methods to isolate sEVs according to their study design.

## 4 MESENCHYMAL STEM CELL‐DERIVED sEVs IN REGENERATIVE AND ANTI‐INFLAMMATORY THERAPIES

Mesenchymal stem cells (MSCs) are multipotent cells that reside in many adult tissues. It is well‐recognized that MSCs exert an anti‐inflammatory and regenerative function.^28^ Accordingly, it has been reported that MSC‐derived sEVs held similar therapeutic effects to MSCs as some immune‐modulating factors from MSCs were highly enriched in sEVs.^29^ Therefore, sEVs derived from MSCs are able to treat diseases related to dysfunctional immune reactions, such as autoimmune diseases, graft‐versus‐host disease (GVHD), and inflammatory diseases theoretically.^30^ Compared to MSCs themselves, MSC‐sEVs reduce the risk for teratoma formation and embolization, which are major concerns for stem cell‐based therapy. As one of the most promising sEVs in therapy, a standardized protocol for MSC‐sEV preparation was defined by International Society for Extracellular Vesicles (ISEV) due to the heterogeneity of MSCs.^31^

**FIGURE 1:**
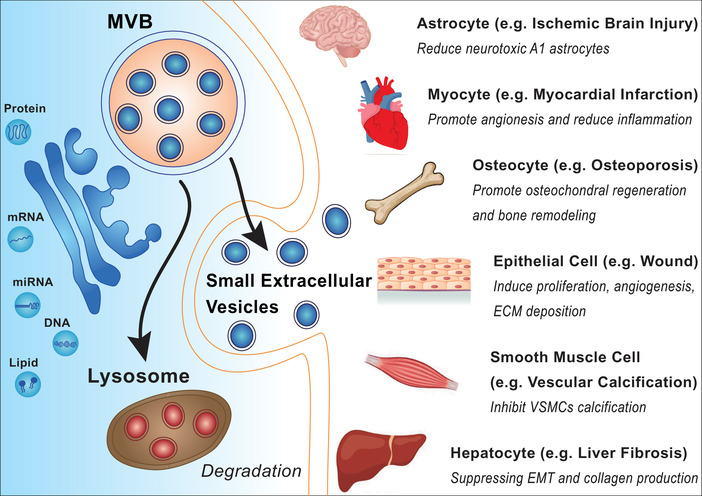
Application of MSC‐derived sEVs in regenerative and anti‐inflammatory therapies. MSC‐derived sEVs target various organs and cells such as astrocytes, myocytes, osteocytes, epithelial cells, smooth muscle cells, and hepatocytes

MSC‐sEVs therapy reduced the pro‐inflammatory cytokine in patient's PBMCs and improved the clinical GVHD symptoms through interleukin‐10 (IL‐10), transforming growth factor β (TGF‐β), and human leukocyte antigen (HLA‐G).^32^ A majority of the literature demonstrated that MSC‐derived sEVs showed protective activity in several diseases such as myocardial infarction,^32^ stroke,^32^ fibrosis,^33^ and ischemia.^34^, ^35^ For instance, MSC‐sEVs reduced infarct size by pro‐angiogenesis and anti‐inflammation as the mediator of tissue repair in myocardial ischemia/reperfusion injury mouse model.^36^, ^37^ Intra‐articular administration of MSC‐sEVs improved the repair of osteochondral defects in rat model.^38^ By suppressing both the EMT of hepatocytes and collagen production through TGF‐β1/Smad pathway, sEVs derived from MSCs could ameliorate liver fibrosis in CCl_4_‐induced liver injury model.^39^ In addition, MSC‐sEVs showed capability in promoting tissue regeneration. sEVs from MSCs could enhance cutaneous wound healing by inducing epithelial cell proliferation through Wnt/β‐catenin signaling pathway.^40^ In addition, MSCs‐sEVs promote the angiogenesis in skin lesions by inducing growth factors through activating AKT/ERK/STAT3 signaling pathways.^41^ MSCs‐sEVs also exhibited a therapeutic potential in traumatic brain injury by protecting neurons and promoting axonal growth via SNARE complex.^42^ In general, these studies displayed the broad therapeutic effects of MSC‐derived sEVs (Figure 1).

## 5 sEVs IN CANCER THERAPY

### 5.1 Cancer immunotherapy by sEVs‐based vaccines

**FIGURE 2:**
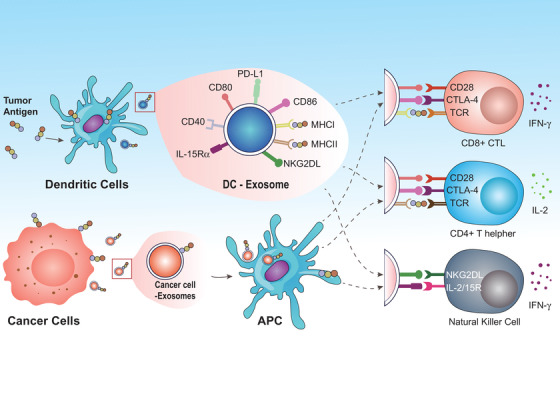
sEVs‐based vaccines in cancer therapy. sEVs derived from dendritic cells express functional MHC‐I, MHC‐II, and T‐cell costimulatory molecules to present antigen and activate T cells as well as natural killer cells. Cancer cells‐derived sEVs express tumor antigens and can be uptake by APCs, such as dendritic cells, therefore initiating immune response

Many evidences suggest that sEVs could be used as novel cancer vaccines to initiate immune system to identify and diminish cancer cells through antigen‐presenting cells (APCs)^43^ (Figure 2). Dendritic cells (DCs) are APCs that can be activated upon stimulus. The crosstalk between DCs and T cells triggers an immune response by specific antigens.^44^ It has been proven that DCs‐sEVs expressed functional MHC‐I, MHC‐II, and T‐cell costimulatory molecules such as CD86. Tumor peptide‐stimulated DCs‐sEVs initiated cytotoxic T cells (CTLs) in vivo and inhibited tumor growth in a T cell‐dependent approach in a mouse model.^45^ Besides antigen presentation, DCs‐sEVs directly triggered natural killer cell proliferation and activation through surface proteins such as NKG2D ligand and IL‐15Rα.^46^

In many respects, sEVs derived from cancer cells are like APCs because of the expression of the tumor‐associated antigen. Interestingly, cancer cells‐sEVs not only have antigen‐presenting molecules (MHC class‐I, HSP), but are also enriched with various tumor antigens such as HER2/neu, melan‐A, TRP, gp100,^47^ CEA,^48^ and others. Therefore, the uptake of cancer cells‐sEVs by DCs induced cross‐presentation to CTLs as a source of an antigen or antigens. Immunization of mice with DCs pulsed with cancer cell‐derived sEVs exerted CD8^+^ T cell‐dependent antitumor effects in mouse models.^49^ It is noteworthy that sEVs may be better than other forms of antigen such as whole‐cell lysates or soluble antigen owing to the advantageous delivery in the form of sEVs^50^ through molecules such as Mfg‐E8 (lactadherin),^51^ integrins,^52^ tetraspanins, and others.^53^ In B16BL6 melanoma mouse model, investigators established a sEVs‐based tumor antigens‐adjuvant co‐delivery system. B16BL6‐sEVs containing tumor antigens could induce B16BL6‐specific T‐cell response and suppress tumor growth.^54^

Peritoneal cavity fluid‐sEVs from cancer patients also showed antitumor effects by inducing DCs to activate T cells in an MHC I‐dependent manner therefore killing cancer cells. Besides, sEVs derived from ascites triggered the increase of IFN‐γ by peripheral blood lymphocytes.^47^, ^55^

### 5.2 sEVs cargo loading for cancer therapy

The natural characteristics of sEVs make them an ideal cargo for delivering drugs, miRNAs, siRNAs, and proteins in cancer therapy. Because of the protective structure of sEVs, certain chemicals and drugs can be loaded on sEVs. In that case, the target biochemical could be distributed to a broad range of biological fluids with a longer circulating time and possibly better efficacy. Immature DCs‐sEVs loaded with Doxorubicin (Dox) displayed a high efficiency in targeting breast cancer cells and delivering Dox, thus inhibiting tumor growth without overt toxicity.^56^ In a zebrafish model, sEVs significantly increased the absorption and cytotoxicity of doxorubicin and paclitaxel, both of which are anticancer drugs, in brain cancer.^18^

The small interfering RNAs (siRNAs) represented a powerful strategy for inhibition of gene expression and were applied to gene therapy.^57^ sEVs‐delivered siRNA caused effective posttranscriptional gene silencing in recipient cells and also exhibited a therapeutic potential in various cancers.^58^, ^59^ Restore of the expression level of tumor‐inhibitory miRNAs in cancer cells are identified to effectively suppress cancer progression.^60^ B cells infected with Epstein‐barr virus (EBV) that encodes miRNAs could secrete sEVs containing miRNAs. Then sEVs were transferred to target cells and repressed the target immunoregulatory genes in EBV‐associated lymphomas.^61^ sEVs derived from miR‐146a overexpressing HEK293 cells inhibited cell growth in prostate cancer by suppressing target gene.^62^

Besides delivery of drugs and nucleic acids, proteins are in great attention as they serve crucial functions in essentially all biological processes. sEVs can not only deliver tumor antigens^63^ as discussed above, but also apoptosis‐related proteins such as caspase‐1,^64^ peptides,^65^ and other cancer‐associated proteins into target cells for cancer therapy. When donor cells harbor a dominant‐negative mutant of Survivin (Survivin‐T34A) that blocks the inhibition of apoptosis, the sEVs increased the apoptotic cell death and enhanced the Gemcitabine sensitivity in pancreatic adenocarcinoma.^66^ More recently, sEVs from CAR‐T cells showed promising therapeutic efficacy with low toxicity.^67^

### 5.3 Clinical trials of sEVs‐based therapy

In view of the promising results achieved both in vitro and in vivo, sEVs‐based therapy has been considered to be an encouraging approach for cancer treatment. Quite a few early phase clinical trials have already been performed or are ongoing (Table 1). The first phase I sEVs‐based clinical trial was launched in metastatic melanoma patients through vaccination with autologous DCs‐sEVs and loaded with the MAGE tumor antigens for 4 weeks. The outcome confirmed the feasibility and safety of DC‐derived sEVs‐based vaccination in melanoma patients for the first time.^68^ Nearly the same time, another phase I trial of DCs‐derived sEVs immunotherapy was performed on nonsmall cell lung cancer (NSCLC) patients. The vaccination was also well‐tolerated and exhibited an activated immune response in some patients.^69^

**TABLE 1:** Clinical trials of sEVs‐based therapy

Year (Completed/Updated)	Disease	Phase	sEVs source	Status	Reference
2005	Metastatic melanoma	I	DCs	Completed	^62^
2005	NSCLC	I	DCs	Completed	^63^
2008	Colorectal cancer	I	Autologous ascites	Completed	^49^
2016	Advanced NSCLC	II	DCs	Completed	^65^
2018	Malignant glioma	I	Glioma cells	Completed	NCT01550523
2019	Head and neck cancer	I	Grape	Ongoing	NCT01668849
2019	Colorectal cancer	I	Plant	Ongoing	NCT01294072
2020	Metastatic pancreas cancer	I	Mesenchymal stromal cells	Ongoing	NCT03608631

Abbreviation: NSCLC, nonsmall cell lung cancer.

Evidence suggested that sEVs isolated from melanoma patients’ ascites delivered Mart‐1 and tumor antigens to DCs for cross‐presentation to CTLs.^47^ Thus, ascites‐derived sEVs combined with GM‐CSF treatment were used in a phase I clinical trial for colorectal cancer. The study indicated that ascites‐derived sEVs in combination with GM‐CSF are safe, nontoxic, and could induce antitumor CTLs response.^55^ It has been reported that sEVs treated with IFN‐γ induced immune activation and tumor suppression.^70^ Based on these, a phase II trial of IFN‐γ‐DC‐derived sEVs was performed on advanced NSCLC patients. The result showed that IFN‐γ‐DC‐derived sEVs boosted NK cell‐mediated antitumor immunity.^71^

A recently completed phase I clinical trial was performed in malignant glioma patients. Glioma cells‐sEVs were loaded with an antisense molecule drug and re‐implanted to patients. These sEVs were expected to stimulate the immune system and induce a T cell‐mediated antitumor response. The result has not been published yet and is of great interest (NCT01550523).

Some other clinical trials concerning plant‐origin sEVs in cancer therapy are currently being investigated. One clinical trial is designed to determine the effects of grape‐derived sEVs‐like nanoparticles on reliving oral mucositis and related pain in head and neck cancers patients (NCT01668849). Another phase I clinical trial was using plant‐derived sEVs to deliver curcumin in colorectal cancer. The efficacy of curcumin‐loaded sEVs in the treatment of cancer patients is going to be detected (NCT01294072). Recently, a group in M.D. Anderson Cancer Center found that sEVs carrying siRNA specific to oncogenic Kras^G12D^, a common mutation in pancreatic cancer, suppressed cancer progression in pancreatic cancer mouse models and significantly increased overall survival.^72^ Based on this, they are conducting a phase I clinical trial regarding the sEVs‐based therapy in metastatic pancreas cancer with Kras^G12D^ Mutation (NCT03608631).

All the above studies (both completed and ongoing) suggested the fact that sEVs‐based therapies are safe in a clinical setting, whereas their therapeutic efficacy is still under evaluation.

## 6 STRATEGIES FOR ENGINEERING sEVs

To better translate sEVs‐based treatments to clinical setting, there are still many obstacles needed to be overcame. Such as how to increase the carrying capacity of drugs, RNAs, and proteins on sEVs and how to improve the specificity of sEVs to target cells. Therefore, various strategies were designed to bioengineer sEVs.

### 6.1 Origins of sEVs and factors promoting the production

The origin of sEVs determines its natural characteristics. Immature DCs‐derived sEVs are low immunogenic because of the lack of surface markers such as MHC‐I, MHC‐II, CD40, and CD86. sEVs from MSCs are able to mediate the immunosuppressive effect.^73^ CD8^+^ T lymphocytes‐derived sEVs express cytotoxic molecules such as granzyme B and perforin, therefore harboring the cytotoxic potential.^67^ HEK293T cells and BJ cells are easy to expand and stable for transfection. Therefore, they are capable of large‐scale production of sEVs. Besides cells, agricultural products such as plants, fruits, and milk can be used as other sources for isolating sEVs that are easy to produce and safe to use by not activating host immune response.^23^, ^74^

Several factors are found to improve the production of sEVs, such as increased intracellular calcium level^75^; external stress including thermal stress,^76^ anoxia,^77^ radiation,^78^ and lower pH^78^; cytoskeletal blocking^79^; drugs such as sitafloxacin, forskolin, fenoterol, nitrefazole, and pentetrazol^80^; and expression of certain genes, for instance, nSMase2,^81^ CD9,^82^ and HIF‐1α.^83^ It is noteworthy that immortalized cell line is more stable and convenient than primary cells in preparing sEVs. A myc‐mediated immortalization of MSCs was developed to produce sEVs and exhibited therapeutic effect in cardiovascular diseases.^84^ Immortalized adipose‐derived Mesenchymal stem cell line (ATCC^®^ SCRC‐4000) has currently been used and proposed as one of the strategies for scale up and mass production of sEVs in a reproducible manner.

### 6.2 Loading nucleic acids into sEVs

Electroporation is the most common way to load RNAs into sEVs. TRPP2 siRNA was loaded into 293T‐derived sEVs by electroporation, and then the siRNA loaded sEVs‐targeted FaDu cells, a cell line of human pharyngeal squamous cell carcinoma, leading to the inhibition of the EMT.^85^

Some transfection reagents have also been used in RNA loading. The purified sEVs could be incubated with siRNA mixed with lipofectamine at room temperature for 30 min, followed by three to five times wash and ultrafiltration. When culturing the recipient cells with engineered sEVs, the uptake of sEVs enabled exogenous siRNA transferring to recipient cells.^58^

There are other novel ways to actively packaging nucleic acids into sEVs. One is binding specific RNA sequences to proteins and the RNA packaging device in sEVs producing cells could package specific mRNAs into sEVs. By using this system, the therapeutic mRNA that significantly reduces the neuroinflammation and neurotoxicity could be successfully delivered to the brain in the Parkinson's disease mouse models.^86^ The conserved sequence of sEVs‐enriched RNAs (eRNAs) carries a certain sequence that targets RNAs into sEVs as a *cis*‐element. Therefore, the use of eRNAs is another approach to deliver RNA into sEVs.^87^ A couple of proteins such as MVP, GW182, AGO2, and Myoferlin were reported to be associated with the delivery of nucleic acid sequences into the sEVs. However, the specific ability for packaging remains to be explored in the future.^88^

### 6.3 Loading proteins into sEVs

One practical method is to overexpress the proteins of interest in donor cells by transfecting the vectors containing the specific gene. Then donor cells produce the proteins encoded by the inserted genes and during the natural packaging processes, the proteins are going to be secreted into sEVs.^89^ However, the potential cytotoxicity to donor cells, the complicated interactions in cells, and the low loading efficiency are the main concerns to make that. Hence, several efforts have been made to promote the loading efficiency of target proteins into sEVs.

The fusion of target proteins with the constitutive protein in sEVs and the specific modification of target proteins are valuable ways to engineer sEVs. A group of proteins that are constitutively expressed on sEVs, such as CD9, CD63, and TSG101,^90^ can enhance the specificity of target proteins. Other confirmed proteins including HIV‐1 Nef (mut),^91^ VSVG,^92^ lactadherin C1C2 domain,^93^ LAMP2B,^94^ and PDGFR TM domain^95^ are demonstrated to package certain proteins into or on the surface of sEVs.

It has been demonstrated that specific modifications of target protein contribute to protein transport. Ubiquitination of target proteins at the C‐terminus led to a 10‐fold increase of expression in sEVs with biological function.^96^ In addition, myristoylation or palmitoylation tag or a transmembrane motif fusion on the N‐terminus of target proteins enabled the fusion proteins to target the plasma membrane.^97^ A major concern of the modifications on proteins is the potential alteration of protein function, especially the ubiquitination modification that may result in the degradation of the target protein by the proteasome.

Several mechanical methods were tried to load proteins to sEVs: sonication, mechanical extrusion, saponin permeabilization, incubation at room temperature, and repeated freeze‐thaw.^19^ Among these, the first three ways displayed high efficiency in vitro. When using mechanical methods, it requires high sEVs purification and careful maintenance of the integrity of sEVs and biological activity.

## 7 CHALLENGES FOR DEVELOPING sEVs‐BASED THERAPY

Small EVs are an exceptionally qualified biological vehicle for clinical therapies. However, there are still several challenges and obstacles needed to be overcome. The first problem to be solved is how to generate a sufficient number of sEVs. Therefore, developing a scalable and reproducible protocol is of great importance. In addition, the criteria for purification and storage of sEVs, as well as the quality analysis, are necessary to attain compliance with good manufacturing practice guidelines.^98^ sEVs from nucleated cells present some level of risk for horizontal gene transfer because it is unpredictable that which cells already harbor dangerous DNA. Bioengineering of red blood cells that do not have nuclei is a potential safer alternative. There are several parameters require to be considered when treating a patient with therapeutic sEVs: dose of sEVs, dosage regimen, and way to administration. These parameters are hard to define as previous studies were mainly performed by empiric therapy without a standard.^99^ In that case, an appropriate way to assess the biological effect caused by sEVs in vitro and in vivo is of the essence. Besides these, optimizing approaches to enhance the sEVs loading efficiency determine the therapeutic activity. Meanwhile, further work still remains to be accomplished to elucidate the exact mechanisms of the interaction between sEVs and targeted cells.

## 8 CONCLUSION AND PERSPECTIVE

Small EVs have gained increasing attention in understanding disease etiology. The robust biocompatibility enables sEVs to be optimum vehicles for delivering chemicals and other therapeutic molecules. Meanwhile, some natural characteristics of sEVs obtained from parental cells make it a promising novel approach for treating a diverse variety of diseases. Because almost all the cells produce sEVs, the donor cells could be selectively chosen based on the clinical needs.

Even though sEVs‐based therapy showed an extensive application prospect, further studies are still needed for in vivo studies and clinical trials. Besides the challenges we discussed above, it is also urgent to investigate the exact components from sEVs that may facilitate the progress of diseases. In the future, more engineered methods are needed to remove the disease‐supporting content from sEVs and enhance the cargo loading efficiency of sEVs. Nanosized sEVs may have immense applications in future clinical therapies.

## AUTHOR CONTRIBUTIONS

Yeye Guo wrote the initial draft of the manuscript. Huaishan Wang, Lili Huang, Lingling Ou, Jinjin Zhu, and Shujing Liu provided the resources for this work. Xiaowei Xu conceptualized the study, edited the manuscript, and provided the funding for this work.

## CONFLICT OF INTEREST

The authors declare no conflict of interest.

## ETHICS APPROVAL

Not applicable.

## Data Availability

Not applicable.
